# Role of Genetically Modified Microorganisms for Effective Elimination of Heavy Metals

**DOI:** 10.1155/2024/9582237

**Published:** 2024-11-09

**Authors:** Shashi Kiran Misra, Ajay Kumar, Kamla Pathak, Girish Kumar, Tarun Virmani

**Affiliations:** ^1^School of Pharmaceutical Sciences, CSJM University 208024, Kanpur, Uttar Pradesh, India; ^2^Faculty of Pharmacy, Uttar Pradesh University of Medical Sciences Saifai 206130, Etawah, India; ^3^Amity Institute of Pharmacy, Amity University, Greater Noida, Uttar Pradesh, India

**Keywords:** anthropologic processes, bioaccumulation, biogeochemical cycle, bioremediation, biosorption, heavy metals

## Abstract

Heavy metals are lethal and hazardous pollutants for the ecosystem owing to their virtues including acute toxicity, prolonged persistence, and bioaccumulation. These contaminants are not only a threat to aquatic/terrestrial biota but also pose serious health issues to humans. Natural and anthropologic processes consistently upsurge heavy metal concentration beyond acceptable limits and mobilization and hence disturb biogeochemical cycles and the food chain, although several conventional strategies including adsorption, chemical precipitation, ion exchange, and membrane separation methods are being employed for the removal of these lethal heavy metals from the ecosystem but failed due to lower efficiency rates and high application charges. The current scenario highly demands advanced biosorption or bioaccumulation processes that slow down heavy metal mobilization within the acceptable limit in the ecosystem. Genetically modified microorganisms (GMMs) with desired features are developed through interdisciplinary participation of genomics, molecular microbiology, and bioinformatics that have more potential to bioremediate heavy metals than the native microbes from polluted ecosystems. The study focuses on different sources of heavy metals, their impact on the ecosystem, and the bioremediation of toxic heavy metals via GMMs.

## 1. Introduction

Revolution in industries is highly demanded for the strengthening of economic conditions in both developed and developing countries. Heavy industrialization, hasty urbanization, and mechanization are continuously polluting our zone of life on Earth, that is, the biosphere [1].

The rate of mobilization of heavy metals has been accelerated day by day via anthropogenic causes such as mining, agricultural activities, industrial discharge, and smelting. Ignition of fossil fuels and exhaustive usage of phosphate composts (pesticides) constantly release these lethal heavy metals in the network, pollute food chains, and create different health issues in not only human beings but also other creatures [2, 3]. Despite natural existence, inevitable human traits or anthropomorphic causes are potent sources that offer toxic concentrations of heavy metals to the environment. Several eastern and western countries including India, Germany, China, Hong Kong, Greece, and Italy have been vigorously affected by the consequences of heavy metal–polluted groundwater systems [4]. Although the slow depletion of heavy metals is mediated naturally via plant uptake, leaching, and erosion, their indiscriminate discharges into water and soil created critical pollution levels worldwide. These released heavy metals are difficult to wreck into nontoxic form, hence creating unhealthy effects on the ecosystem (plants, animals, and humans). Heavy metals including chromium (Cr), selenium (Se), lead (Pb), cadmium (Cd), mercury (Hg), arsenic (As), and nickel (Ni) are reported to be mutagenic, cytotoxic, carcinogenic, and deadly even at very low concentrations (~1 *μ*g) [5]. These heavy metals are accepted as major contaminants by virtue of their remarked density, excessive toxicity, and nonbiodegradability. The United States Environment Protection Agency (USEPA) has alarmed the prevalence of major eight harmful heavy metals, that is, Hg, Cd, zinc, Pb, Ni, As, copper (Cu), and Cr in the soil and groundwater systems of the environment [6].

Discrete groups of heavy metals retain high atomic weight/number with high densities. They are nonbiodegradable and hence considered lethal in very low concentrations in the living entities. Due to anthropological activities (industrial and agricultural), mining, and automobile exhaust, most of the heavy metals get deposited as toxic chemicals or fumigants and pose a threat to humans by causing fatal ailments and mutations in plants. Coordination chemistry has classified these heavy metals in Category B which are considered toxic and nonessential trace elements. Literature envisages that naturally occurring metals have atomic numbers of more than 20 with high elemental density, that is, 5 g/cm^3^ [7].

US-governed CERCL, a Comprehensive Environment Response Compensation and Liability Act, has proclaimed the maximum accepted limit of these metals in water systems [8].  Table 1 compiles the permissible limits of heavy metals. Beyond these limits, heavy metals are recommended as human life-threatening pollutants and are concerned with several complications related to atherosclerosis, neurological disorders, and myriad cancers [11]. In developing countries, humans are extremely exposed to these noxious heavy metals either by ingestion (food/drink), dermal absorption, or inhalation (fumes) at their workplaces. For instance, workers and laborers engaged in mining and industrial operations continuously inhale dust enriched with toxic metals. People who extract gold remain exposed to Hg vapors during the amalgamation process. Welders are also reported to have traces of Cr, Pb, Cd, and Ni in the blood sample due to inhalation of welding fumes. Tobacco and cigarettes are also common sources for the dissemination of Cd and other heavy metals [12]. Various anthropological sources and permissible limits of heavy metals are enlisted in Table 1. Further, heavy metal–contaminated terrestrial (plants and animals) and aquatic (fish) food chains pose serious threats either by exhibition of additive, antagonistic, or synergistic interactions to the consumers. Literature envisaged that six heavy metals including Pb, zinc, Cr, Hg, Cd, and Cu cause the most acute toxicity in aquatic fauna and flora. Invertebrates are more susceptible to these heavy metals than vertebrates. Cu and Hg are more lethal and have crucial adverse effects on aquatic vertebrates [13]. Similarly, As (III) and (V) are hazardous inorganic forms of heavy metals and exist in different categories, that is, metalloids (As0), arsenites (trivalent, AsIII), and arsenates (pentavalent, AsV). As it reaches the water system, it causes serious ailments such as arsenicosis, cancers, hyperkeratosis, neurodegeneration, and other vascular diseases [14, 15]. Trivalent As is more lethal, has higher solubility, and is more movable compared to pentavalent As. Microorganisms occur in soil, which metabolizes As into volatile components [15]. Thus, the current scenario requires innovative research-based newer technology that slows down heavy metal pollution within the acceptable limit in the ecosystem. In this prospect, myriad physical (electrokinetic remediation, vitrification, thermal desorption, and vapor stripping), chemical (precipitation, ion exchange, membrane filtration, coagulation–flocculation, and electrodialysis), and biological strategies (biosorption, anaerobic digestion, phytoremediation, and biotransformation) have been employed to remediate toxic metal-polluted soil and water [16].

However, these approaches face issues related to cost, efficiency, and failure in execution and are unfit for the separation of dissolved heavy metals in water [17]. To resolve the above downsides, upgraded integrated treatment strategies such as chemical biological remediation, electrokinetic microbial, and phytoremediation are welcomed, and those are economic, effective, versatile, and ecofriendly [18, 19]. Alternatively, biological or microbial-based strategies, that is, bioaccumulation and biosorption (adsorption), are successful processes for bioremediation of these toxic heavy metals from the contaminated sites and reestablishment of the natural condition of the ecosystem (soil and water systems).

Microbes by their metabolic activity degrade environmental pollutants and heavy metals [20]. Microbial bioremediation is involved in recovering and removing hazardous heavy metals containing e-waste by following environmental protocols and legislation [21]. Graphene and its derivatives have been explored for adsorption and removal of Cd heavy metal from biota owing to its high adsorption efficiency. It is considered superior to activated carbons and carbonaceous adsorbents for the purification of water systems from heavy metals [22]. However, the low concentration and nature of heavy metals in the ecosystem may cause modifications in plants and microbial communities such as inhibitory actions and chemical changes via blockage of functional groups [23]. Recent studies elucidated that these heavy metals can accelerate the transference of antibiotic-resistance genes in microorganisms, particularly in sludge bacteria [24]. These microbes effectively remediate heavy metals either by bioaccumulation (actively) or bioabsorption (passively) process. There is a process of conjugation between functional groups adorned on lipid/polysaccharides present in the cell walls of microbes and metals. Eukaryotic microbes such as fungi (species of *Aspergillus*, *Penicillium*, and *Rhizopus*) are extensively utilized to remove heavy metals from polluted water systems via the process of bioadsorption [25]. The review provides insights into different sources of heavy metals, their impact on the ecosystem, and the significant roles of genetically modified microorganisms in bioremediating toxic heavy metals.

## 2. Sources of Heavy Metal in Biosphere and Processes of Detection

Among versatile natural sources for the occurrence of heavy metals in the environment, rocks (sedimentary, metamorphic, and magmatic rocks) are the major ones. Molten rocks or magma contains a high concentration of chemical elements including heavy metals that are transported to the soil, the earth's surface. Further, weathering, erosion, and weather change cause the physical destruction of rocks that get fragmented into sediments containing excess levels of Ni, manganese, barium, cobalt, Pb, lithium, Cu, and zinc. Alhogbi, Al-Ansari, and El-Shahawi [26] researched to quantify trace metals in vegetables grown in pesticide-enriched soil near Jeddah, Saudi Arabia. Collected samples (soil, irrigation water, and cultivated crops) were analyzed via an inductively coupled plasma–optical emission spectrometer. The level and uptake of iron, Cr, and Pb in soil samples were detected as distinctively higher as recommended by the World Health Organization and Food Administration Organization. These trace elements were not alarming in the analyzed samples of water and vegetables [26]. Several anthropogenic sources including human activities are mining, electroplating, smelting, usage of fertilizers, and pesticides, and the release of biosolids (livestock manure, sewage, and sludge) disturbs the geochemical cycle and accumulates metals in soil/water systems. Heavy metals (Cd and zinc) are frequently incorporated in phosphate fertilizers to improve crop productivity and pesticides containing Pb, As, and Hg to protect against crop invaders. These toxic metals are infiltrated into the water system, hence contaminating the ecosystem [27]. Excessive industrialization and manufacturing units discharge toxic heavy metal wastes abundantly that are usually transported through the soil matrix and exposed to the flora, fauna, and humans. Most of the metal wastes are dissolved in the water system, and undissolved ones remain dispersed on the soil surface and severely affect the agriculture system and food chain. Several factors, that is, soil moisture, pH, water, clay content, temperature, and nature of heavy metal influence mobility, solubility, adsorption, accumulation, and toxicity in the soil system [3]. Heavy metals/metalloids polluted soil is carried out in the various water systems such as rivers, ponds, and lakes via sewage, industrial effluents, and landfill leachates. Aquatic biotas are heavily influenced by excessive discharge of toxic metals (Hg, aluminium, As, Pb, iron, Ni, zinc, Cd, and barium).  Table 1 displays diverse anthropological causes of heavy metal contaminants in the environment. Apart from soil and water systems, the atmosphere is also affected by heavy metals [28]. Natural sources, that is, mineral dust, forest fires, volcanic eruptions, and industrial processes (cement factories and metal smelters) release volatile metals (Se, As, Hg, and antimony) in the gaseous system of the atmosphere, although few metals such as Pb, zinc, and Cu remain in particulate form in the atmosphere [29]. Agriculture-based products are essential for the survival of human life and are produced by the applications of organophosphorus chemicals and pesticides to enhance the yield and growth of crops. These include myriad hazardous heavy metals which are exposed to humans via the intake of vegetables, cereals, and fruits [30]. In most developing countries, most of the population is below the poverty line (BPL) and cultivates crops near refused grounds which are enriched with waste effluents and toxic metals. Thus, these populations face fatal health issues and remain ignored due to poor economic conditions. Awino et al. [31] investigated the extent of bioaccumulation of heavy metals in the crops grown in Uganda (Mbale dumpsite) and studied the higher extent of accumulation of Pb and cobalt into the grown crops of *Amaranthus cruentus* and *Zea mays* that were lethal to consumers, according to the Food and Agriculture Organization. Hence, there should be public awareness events and remedies to reduce the risk of health issues in that community whose survival relies on such cultivation crops [31]. Several methods such as atomic absorption spectrometry (AAS), laser-induced breakdown spectrometry, inductively coupled plasma–mass spectrometry, and atomic fluorescence spectrometry are utilized for the quantification of heavy metals from polluted soil and water systems [32].  Table 2 compiles several analytical tools utilized to detect heavy metals in polluted soil, water, and bioaccumulated plants. Molecular biology is also employed to understand heavy metal–induced stress, chemical form, uptake, spatial distribution, translocation, and accumulation in plants [40].

## 3. Heavy Metals: Impact and Toxicity in the Environment

These elements (heavy metals) upset cellular organelles and their vital components (mitochondria, endoplasmic reticulum, lysozyme, cell membrane, and nuclei) and physiological enzymes. After interacting with cell components, metal ions impair DNA and proteins; consequently, conformational changes mediate variation in the cell cycle and cause apoptosis. The report says that heavy metals can provoke gene alteration through epigenetic changes in chromatin that impact adverse effects and diverse ailments. For instance, Cr (VI) mismatches repair gene *MLH1*, resulting in the overexpression of histone methyltransferase G9a, which is accountable for attaching posttranslational methylation to H3K9, a biomarker of lung carcinoma [41]. A few metals such as Cd, Pb, As, Cr, and magnesium generate free radicals that induce oxidative stress and result in carcinogenicity which are compiled in Table 3. These systemic toxicants raise several health issues at very low concentrations. They are neither broken down nor biodegradable. However, they exert specific biochemical and physiological reactions in humans, animals, and plants. Some of them are detoxified by microorganisms present in soil and aquatic systems. Heavy metals are key components for regulating enzymes and controlling physiological redox reactions in the body [54]. For instance, Cu, an essential cofactor, is involved in oxidative stress-mediated metalloenzymes such as cytochrome oxidase, monoamine oxidase, superoxidase dismutase, ferroxidase, peroxidase, and catalase. These enzymes are associated with carbohydrate metabolism, hemoglobin formation, biosynthesis, and crosslinking of catecholamine, collagen, keratin, and elastin [55]. On the contrary, the transition between Cu (II) and Cu (I) is potentially toxic due to the generation of free radicals (hydroxyl and superoxide) that leads to several adverse effects including cellular damage (Wilson disease) [56]. As is highly used in industrial and agricultural sectors for the synthesis of dyestuffs, wood preservatives, insecticides, weedicides, and fungicides. Its significant applications in veterinary medicine are observed for the suppression of tapeworms in domestic animals and filariasis in dogs [57]. Traditionally, As has been used in the design of different pharmaceuticals to mitigate syphilis, trypanosomiasis, yaws, and amoebic dysentery. Inorganic As is an approved human carcinogen and is lethal for the bladder, liver, kidney, skin, and lung. According to the current guidelines of the WHO, the maximum safe concentration of As is below 10 *μ*g/L [58]. Recently, the USFDA has approved As trioxide as a potent anticancer for the management of acute promyelocytic blood cancer (leukemia) owing to the induction of apoptosis in leukemic cells [59]. Naturally occurring Pb (bluish-grey substance) is found in the upper crust of the earth. Further, anthropogenic processes such as mineral mining, burning of fossil fuels, manufacturing (Pb-acid batteries, paints, metallic products, ammunition, and shield X-rays), and industrial waste contribute to a significant number of heavy metals in the environment. Several reports on Pb poisoning exposed the presence of higher levels of antioxidant enzymes such as glutathione peroxidase and superoxide dismutase in erythrocytes that mediate programmed cell death, DNA damage, transcriptional activation of stress genes, and activation of caspase-3 in humans [60]. Hg, another crucial heavy metal, is a widespread pollutant and environmental toxicant that causes prominent disturbances in the tissues and cells of living organisms. It is exposed to the environment in the form of inorganic/organic mercurial compounds and elemental Hg vapor. Hg is ubiquitous and extensively employed in several chemical syntheses (caustic soda), industries (batteries, switches, and thermostats), and pharmaceuticals (dental amalgams, preservatives, and antifungal compounds) [61]. All forms of Hg are toxic and highly lipophilic and effectively get absorbed through lung tissue linings after exposure. It can rapidly cross cell membranes (placental and blood–brain barrier) and become oxidized and reactive. It shows a very low excretion rate, and the major part accumulates in the vital organs including the liver, kidney, and neurological tissues that mediate gastrointestinal toxicity, nephrotoxicity, and neurotoxicity [62]. The pH of the soil and its composition (organic matter) influence the toxicity of heavy metals. Most of the heavy metals pose a threat to living organisms in acidic and nutrient-deficient mediums [63]. In an acidic medium, heavy metals form free ions with excess protons that saturate metal binding sites. This ionic form is more bioavailable and absorbable which causes toxicity to the existing plants and microorganisms. On the contrary, in alkaline pH, metal ions form soluble hydroxy-metal complexes by replacing protons with other species. Organic matter–enriched soil has a higher capacity to retain heavy metals and does not let them mobile [64].

## 4. Genetically Modified or Bioengineered Microorganisms

Treatment of heavy metal–loaded waste effluents is quite challenging as it is directly concerned with environmental and social activities. Hence, there is a need for such technoeconomical approaches that efficiently curtail heavy metal pollutants from the ecosystem (soil and water).

**Figure 1:**
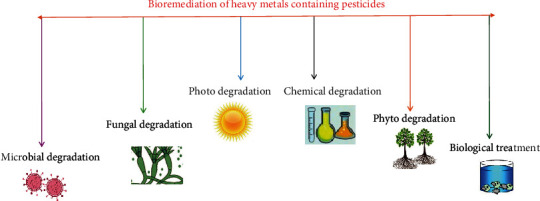
Different traditional bioremediation processes for the removal of heavy metals.

Figure 1 illustrates different bioremediation processes including microbial, fungal, phyto, chemical, and photodegradation. Other methods such as chemical precipitation, adsorption, electrolytic, cementation, solvent extraction, reverse osmosis, and ion exchange are popularly engaged for heavy metal remediation [65, 66]. Genetically modified microorganisms are widely accepted as bioremediate heavy metals, as these are environment-friendly, economical, easy to develop, and their upscalability. Moreover, their structural and molecular modifications make these microorganisms selective, specific, and more efficient in to uptake or binding of heavy metals via overexpression of exogenous DNA or genes on cell surfaces. Genetically modified microorganisms exhibited variable mechanisms to interact with lethal heavy metals including biotransformation, enzymatic processes, formation of metallothionein, and exopolysaccharide [67]. However, several issues including poor competitiveness, variable heavy metal concentration, configuration of cell walls, sorption sites, and ionization may limit the frequent applications of these microorganisms. For instance, the processes of methylation/demethylation, metal complexation, and oxidation are hurdles for efficient bioremediation.  Table 4 compiles a list of various genetically modified microorganisms that significantly uptake heavy metals from the ecosystem.

**Figure 2:**
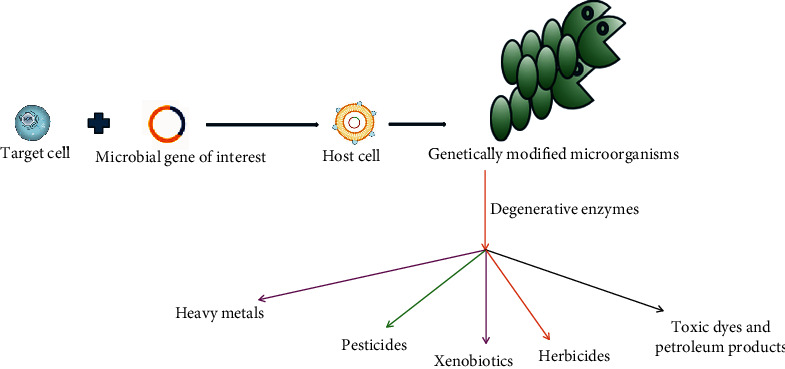
Typical process of development of genetically modified microorganisms explored for pollutant removal from the environment.

Installation cost, removal efficiency, sustainability, and ecofriendliness are a few major concerns of the selected methods for heavy metal decontamination. Biobased or biologically driven technologies such as bioaccumulation and biosorption are employed for heavy metal removal from waste effluents and may resolve the above downsides associated with chemical and physical processes. These biotechnological strategies utilize genetically modified microorganisms and plant biomass to screen out heavy metals from real wastewater effluents. Further, it is considered an ecofriendly and cost-effective approach for withdrawing heavy metals from wastewater [68]. To improve heavy metal uptake, the inner membrane importers of bacteria and fungi (genetically modified) are recombinantly expressed with three major transporters, that is, channels, primary transporters, and secondary carriers [69]. Bioremediation proves a better alternative to ameliorating the undesirable effects of heavy metals generated by anthropogenic actions. The technique is ecofriendly and appropriate for remediating pollutants from the rhizosphere via the involvement of myriad species of microbes including *Acinetobacter*, *Bacillus*, *Microbacterium*, *Corynebacterium*, and *Pseudomonas*. Literature envisages the participation of some plants (*Brassica juncea*, *Solanum lycopersicum*, *Helianthus annuus*, and *Salix viminalis*) to potentially reduce the accumulation of heavy metals in soil.  Figure 2 explains general methods for development of genetically modified microorganisms for bioremediation of heavy metals.

### 4.1. Helical Protein Channels

Helical protein–composed channels are single components that facilitate the passive diffusion of heavy metals. Mostly the process is energy-independent and does not need ATP (nucleoside triphosphate) or proton motive force (PMF) for the translocation of heavy metals [70]. Moreover, the process is governed via a concentration gradient through the inner membrane of genetically modified microorganisms. For instance, TCDB 1.A.8 (transporter classification database) is involved in the bioaccumulation process [71]. These importers or channels belonging to a major intrinsic protein superfamily (TCDB 1.A.8) are reported for efficient uptake of As and Hg metals by various microorganisms such as *Escherichia coli*, *Streptomyces coelicolor*, *Corynebacterium diphtheriae*, and *Saccharomyces cerevisiae*. Literature envisaged that Hg is competently taken up by MeT/P transporters from bacteria, that is, *Pseudomonas* K-62, *Serratia marcescens*, and *Pseudomonas* K-12. Further, Mer superfamily importers (TCDB1.A.72) such as MerF, MerC, and MerE are also found to uptake Hg heavy metals from the environment [72]. The channel importers require zero energy, which is why they are highly utilized for heavy metal bioaccumulating; however, they are reliant on passive uptake. At equilibrium, genetically modified microorganisms can no longer bioaccumulate heavy metals; thus, the design of such GEMs is required that can uptake relatively high concentrations of heavy metals from waste effluents [73].

### 4.2. Secondary Carriers (TCDB 2.A)

These are single-component proteins and may be categorized as uniporters, antiporters, and symporters. Secondary carriers depend on the presence of charge differences across the inner membrane of microorganisms and heavy metals. Thus, PMF is crucial for the translocation of charged cationic heavy metals. Amid symporter belongs to the NiCoT-opsin-G protein receptor superfamily and is highly emerged for bioaccumulating As, Ni, and cobalt [74]. Pho84 symporter from *Saccharomyces cerevisiae* concerns to major facilitator superfamily (TCDB 2.A.1.9) uptakes As. Microorganisms such as *Staphylococcus aureus*, *Helicobacter pylori*, *Rhodopseudomonas palustris*, and *Novosphingobium aromaticivorans* are notified for active import of cobalt and Ni. Hxt7 uniporter from *S. cerevisiae* (sugar portal family; TCDB2. A.1.1) uptakes As [75].

### 4.3. Primary Active Transporter (TCDB 3.A, PAT)

It is a multicomponent protein complex (TCDB 3.A) that contains a transmembrane component and involves cytoplasmic energy coupling ATPase for the translocation of heavy metals. These transporters utilize phosphoanhydride bond hydrolysis, periplasmic solute binding, and hydrolysis of NTPs to pursue the uptake process. Cd has been bioaccumulated by *Lactobacillus plantarum* through MntA/cdtB, primary active transporters, and Cu from Enterobacter hirae via CopA. Li et al. investigated the effect of exposure to different heavy metals (Cd, Co, and Hg) in *Escherichia coli* cells. Phytochelatin synthase gene *PcPCS1* (absorbs heavy metal ions) in *E. coli* Rosetta was explored for removal of heavy metal ions [76]. PAT straightly utilizes chemical energy, that is, cellular ATPase that enforces a high energetic load on the microorganisms. TCDB3. A.1. is another ABC transporters superfamily that has three classes, that is, Type I, II, and ECF. Pathogenic bacteria (Yersinia pestis, causative of bubonic plague) have a high affinity for Ni heavy metal via the *YntABCDE* operon, an ABC active transporter [77]. Some pathogenic microorganisms, that is, *Y. pestis*, *P. aeruginosa*, and *S. aureus* can uptake picomolar heavy metals via solute binding components (approximately 70 Da) through present metallophores such as yersinopine, pseudopaline, and staphylopine, respectively. These metallophores have the efficiency to uptake small concentrations of metals from the human respiratory system and minimize the virulence of pathogens too [78].  Table 5 compiles a few transporters of microorganisms that competently uptake heavy metals from the environment.

## 5. Strategies and Mechanisms Involved to Clean Up Heavy Metals

Microorganisms such as algae, fungi, and bacteria ubiquitously occur in the ecosystem and contribute a vital role in the remediation of environmental heavy metal pollutants. The nature of microorganisms and soil/water properties (pH, presence of organic matter, humidity, presence of ions, colloidal substances, moisture contents, and other organisms) influence the uptake efficiency of microorganisms. Various successful strategies including bioremediation, biosorption, and phytoremediation are reported to remove toxic heavy metals utilizing microbes and biomass.

### 5.1. Biosorption

Biosorption is a biodriven technique that involves alive/dead biomass for detoxifying environmental heavy metal pollutants; hence, it has a crucial role in the biogeochemical cycle. The heavy metals are converted into less harmful contaminants, eventually employed for the mineralization of organics into water, carbon dioxide, and nitrogen gas. Both in situ (biostimulation, biosparging, bioattenuation) and ex situ (land farming, biopile, and bioreactor) techniques are executed for heavy metal bioremediation. The former stimulates indigenous microbes, utilizes genetically engineered microorganisms, and does not alter soil structure, although the geographical location, temperature, soil moisture/type, microbial competition, and the extent of heavy metal contamination are a few aspects to be accounted for in the in situ bioremediation methods. Moreover, high temperature tends to increase the solubility of heavy metals in the soil or water system, hence mobilizing them towards aquatic biota. Sulfhydryl sites present in bacteria play an important role in the adsorption and desorption of Cd ions. Sulfhydryl sites of *Pseudomonas putida* are explored for the detoxification of Cd. Studies revealed that Cd can be linked to both nonsulfhydryl and sulfhydryl sites of bacteria at different growth stages owing to its preeminent tolerant limit of chalcophile metals such as Au, Hg, Cd, and Cu [79].

**Figure 3:**
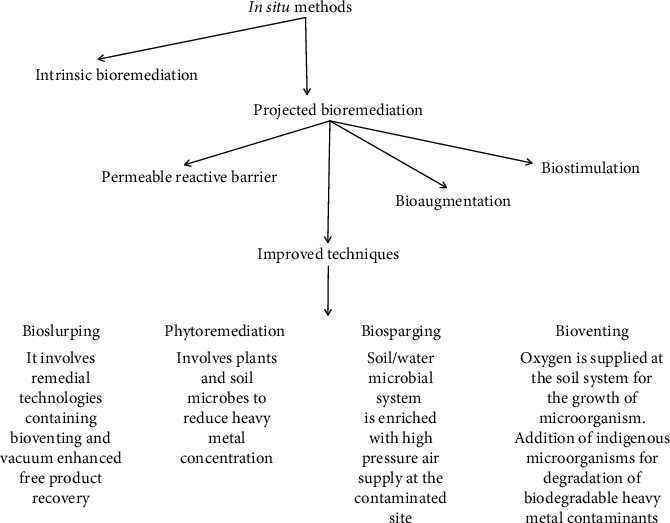
Different in situ approaches involved in bioremediation of heavy metal pollutants.

**Figure 4:**
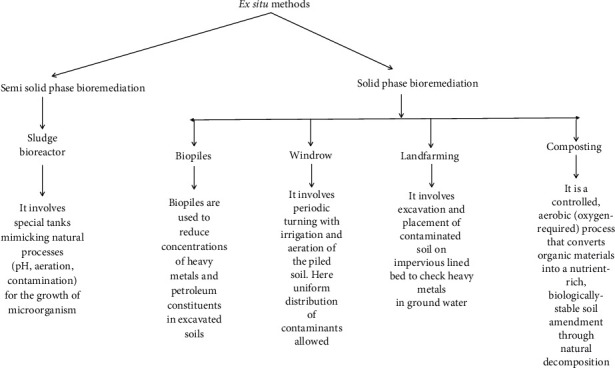
Various ex situ technologies and their specifications.

Figure 3 compiles salient features of a few in situ bioremediation methods [80]. Microbial biosorption is a comparatively efficient and inexpensive way to remove toxic heavy metal pollutants from the environment. The presence of anionic charge on the cell surface facilitates electrostatic interaction with cationic-charged metal ions. For instance, the binding of Cd and Cr metal ions with microbial carboxyl and amine functional groups, respectively, is reported. The greater sorption capacity of microbial cells compared to clay lets them allow for the removal of heavy metals from the contaminated sites. Indigenous microbial species have exceptionally high biosorption efficiency for heavy metals from the contaminated site. For instance, AK1 and AK9 both are heavy metals (Cd, Cu, Pb, Ni, Hg, As, and cobalt) resistant strains of *Pseudomonas* and have been isolated from an As-contaminated aquatic system (Ganga Basin) [80]. Contrary to in situ methods, ex situ techniques are engaged in the transportation of contaminated soil and water samples to the treatment sites. Both solid and slurry phase techniques are utilized for the removal of heavy metals from soil and liquid systems, respectively. Bioreactor, windrow, biopile, and land farming are few ex situ techniques and depend on pH, live/dead biosorbents, the concentration of microbes, and pretreatment process conditions [81]. Heavy metals are removed from synthetic wastewater through a bioreactor where microbial biomass is immobilized in scaffolds such as silica gel and alginates. That provides suitable biosorbants. These kinds of immobilized biosorbants impart sufficient porosity, strength, heat resistance, and physicochemical stability. Species of *Agrobacterium* and *Rhodotorula* have been reported to remove iron metal [82, 83]. Microorganisms including bacteria, fungi, yeast, and algae are nice biosorbents and can absorb heavy metals even in lower concentrations. Their cell surfaces possess different functional groups (amide, amine, carboxyl, carbonyl, and hydroxyl) that bind heavy metals and facilitate their removal from the environment. The presence of characteristic enzymes in their cell structures enables them to be resistant to heavy metal poisoning [84]. Further, some fungi like *Klebsiella*, *Pleurotus*, and *Cephalosporium* utilize heavy metals for their growth and can convert them into less toxic substances [85]. Bacterial species such as *Bacillus*, *Desulfovibrio*, *Geobacter*, and *Pseudomonas* need to be exposed to heavy metal contaminants for the initiation of enzymatic induction and to execute the bioremediation process [86].  Figure 4 displays different kinds of ex situ methods and their mechanisms for the uptake of heavy metal pollutants.

The bioremediation process is governed through various mechanisms such as redox reaction, complexation, adsorption, precipitation, ion exchange, and electrostatic attraction. Microorganisms immobilize metals (iron, Cr, As, and Hg) by redox reactions by virtue of metal oxidation and reduction capacity. Here, insoluble and stationary forms of metals present in sediments are converted into soluble and mobile forms. In most cases, this bioconversion may pose toxic and unhealthy effects to the environment as their increased bioavailability disturbs microbial metabolic processes [87]. Microbial reduction of Hg (Hg II) to more volatile form Hg (0), less soluble Fe (III), and As (V) to more soluble Fe (II) and As (III) is fungal remediation by *Aspergillus niger* that facilitates leaching from sediment or soil.

Debiec-Andrzejewska et al. [88] investigated plant (alfalfa)-microbe (*Ensifer* sp. M14) interactions in natural soil, As rich garden soil, and As polluted soil. *Ensifer* sp. M14 is an effective arsenite oxidizer that augmented 60% biomass with two times high As accumulation without upsetting the natural microbial community after 40 days [88]. Another heavy metal (Hg and Cr) biomethylation process in microbial cells may alter their volatility, mobility, and toxicity, hence important for the process of detoxification. As a result, more volatile dimethyl Hg and alkyl As formed in the microbial cells are removed and evaporated from the soil [89]. Complexation of heavy metal ions with the surface or intracellular components of microbial cells is another way to bioremediate these toxic contaminants. *Klebsiella planticola* and *Pseudomonas aeruginosa* are reported to precipitate Cd through complexation [90, 91]. Bacterial species of *Methanothermobacter*, *Shewanella*, and *Bacillus* have been employed for the reduction of Cr (VI) to Cr (III) and then immobilized in the form of hydroxide-oxide. Mycoremediation comprises live or dead fungal cells to remove environmental contaminants (heavy metals) from different segments. It is an economic process that does not produce harmful waste products in the surroundings. The pervasive presence of fungi in nature enables their excessive utilization in industrial applications. Their advanced cell structures (exclusive morphology and metabolism) are easily adaptable according to environmental conditions. These fungi are the topmost decomposers and impart a role in the nutrient cycle. Further, these microbes can endure under stress conditions including pH, moisture, and nutrients [92]. Mycoremediation completely mineralizes heavy metal pollutants by accumulating them in the fruit bodies of fungi, thus making them unavailable to the surrounding media [93]. However, the process is reliant on the fungal species, its life, the chemical nature of the targeted contaminant, and the media. For instance, *Saccharomyces cerevisiae* sequester approximately 79% Pb and Cd from the polluted soil [94]. Another, *Aspergillus* sp. has been reported to remove out ~65% Cr from tannery wastewater [95]. Literature envisages different mechanisms including adsorption, complexation, and ion exchange for the process of mycoremediations via interactions between the fungal cell wall (chitin, glucan, pigments, proteins, and polysaccharides) and the metal ions [96]. A few mushrooms and wood-decaying species containing white/brown rot fungi (*Termitomyces clypeatus* and *Pleurotus ostreatus*) are utilized for their nutritional and medicinal values. Now, these fungi are explored for metal uptake degradation over their background concentration and accumulate disproportionally in the fruiting body [97]. Species of *Penicillium*, *Aspergillus*, *Agaricus*, *Rhizopus*, and *Trichoderma* are reported for uptake of As, Cd, Cr, cobalt, Pb, and mercurial ions. Similarly, phycoremediation involves the clean-up or removal of marine contaminants via live or dead algae. These are autotrophic organisms and are abundantly found in the marine ecosystem. Brow, red, and green algae (*Phaeophyta*, *Chlorophyta*, and *Rhodophyta*) are reported to bioremediate heavy metal pollutants from the media [98]. Batch or column bioreactors are utilized where complexation between algal proteins comprised of different functional groups (amine, carboxyl, hydroxyl, phosphate, and sulphate) and metal ions is taken place. Moreover, mono and divalent ions (sodium, magnesium, and calcium) present on the cell wall of algae are replaced by metal ions through the principle of ion exchange [99]. Some algae such as *Asparagopsis* (Cd, Cu, Pb, zinc, and Ni), *Chlamydomonas* (Cd, Hg, and Pb), chlorella (Cd, Pb, Ni, and zinc), *Corallina* (Cd, Cu, Cr, and Pb), *Caulerpa* (Cd, Cu, and Pb), *Spirogyra* (As, Hg, Pb, Cd, and zinc), and *Fucus* (Pb, zinc, and Cd) are found to bioremediate metallic pollutants. Few issues are reported with biosorption such as (i) sensitivity for variable pH, ions, and organic compounds present in soil and water waste systems; (ii) nonspecificity for particular heavy metals; (iii) poor capacity and nonreusability of biomass; and (iv) periodically replacement of biomass is required [100].

### 5.2. Bioaccumulation

**Figure 5:**
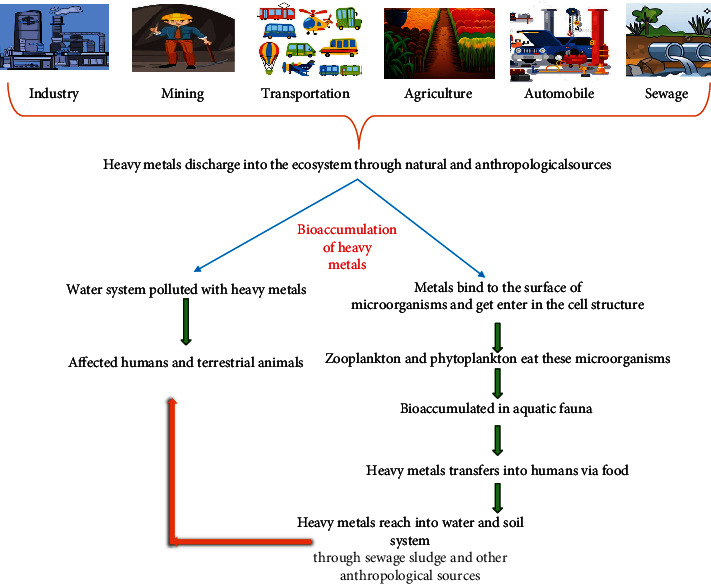
Bioaccumulation and biocirculation of heavy metals in the food chain.

Heavy metals polluting soil are a global issue and have a potential impact on the environment and human health. As, large percentage of land, is reserved for agriculture, that is highly influenced through the application of heavy metal–enriched pesticides, inorganic fertilizers, compost, wastewater irrigation, and sewage sludge [101]. Depending upon the toxicity of these metals, their accumulation in these farmland areas not only pollutes soil, growth, and quality of crops but also poses a threat to the food chain [102]. Contrary to biosorption, bioaccumulation is the direct uptake of heavy metals by living biomass including terrestrial and aquatic biota. The process is metabolic dependent and involves active uptake employing living organisms. However, biosorption is metabolically independent and reliant on passive uptake via dead biomass and microorganisms (bacteria, fungi, and algae). In general, the bioaccumulation process for heavy metal removal is not practically successful, as microorganisms and living organisms are to be exposed to the intake of toxic heavy metals that would cause or interrupt metabolic functions and may lead to death.  Figure 5 portrays how heavy metals are circulated via the food chain.

Microorganisms uptake environmental contaminants (heavy metals) into their intracellular structure through importers. After reaching inside the cells, these heavy metals are sequestered by protein/peptide storage substances. Bioaccumulation capacity is measured to define the concentration of heavy metal uptake in micromoles or milligrams per gram [103]. The process of bioaccumulation faces some challenges such as live host cells, aerobic/anaerobic requisites, nutrients for sustaining/growing biomass, and the requirement of genetically modified microorganisms in the environment [104]. However, bioaccumulation mediates a high concentration of lethal heavy metals inside living organisms including marine creatures, animals, and humans via the food chain leading to severe health issues, noncurable diseases, and even death.  Table 6 compiles heavy metal bioaccumulation capacity by genetically modified microorganisms.

### 5.3. Phytoremediation via Microbes–Plant Symbiosis

**Figure 6:**
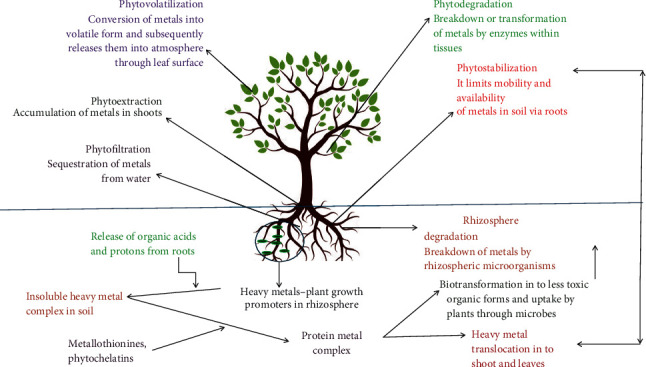
Phytoremediation of heavy metals and plant–microbe interactions.

Phytoremediation utilizes heavy metal remediation from soil, sediments, sludge, and wastewater via plants and associated microbes. Significant uptake of radionuclides, toxic metals, and organic pollutants is very common with this approach [2, 106]. The process is in situ, ecofriendly, cost-effective, and efficient and comprises diverse techniques such as phytoextraction, phytovolatization, phytofiltration, phytostabilizaton, and phytodegradation. Plant metabolic enzymes such as oxygenase and dehalogenase breakdown organic pollutants into simpler forms.  Figure 6 portrays different modes of phytoremediation concerning the removal, biosorption, and bioaccumulation of undesirable contaminants or pollutants from the ecosystem [107]. Plants also contribute to the uptake process via the immobilization process, where heavy metals are absorbed, precipitated, complexed, and reduced by rhizospheric microorganisms [108]. Soil is the upmost sink for metal pollutants, where the concentration of heavy metals varies from 1 to 100,000 mg/kg [109]. Soil microbes or rhizospheric microorganisms detoxify contaminated soil and are termed rhizoremediation. Various rhizospheric microbes impart crucial functions in plant growth and metal uptake, as depicted in Figure 6. This beneficial plant–microbe symbiosis poses successful phytoremediation for bioaccumulation of toxic heavy metals from the environment. Various prokaryotes and eukaryotes are found abundantly near the vicinity of plant roots. Bacterial populations such as *Proteobacteria*, *Pseudomonas*, *Firmicutes*, *Bacillus*, and *Arthrobacter* are involved in the metal uptake process from the contaminated sites. In this series, *Rhizobia* is a significant plant growth-promoting microbe found in the rhizosphere and engages in symbiotic nitrogen fixation [110]. Further, these microbes are highly sensitive to heavy metals and increase the quality of soil. Similarly, *Basidiomycota* and *Ascomycota*, both fungal genera, are found in heavy metal–stressed soil systems [111]. AM (arbuscular mycorrhizal) fungi primarily colonize around contaminated soil, and various intracellular functions are nicely driven by binding with a large number of heavy metals, where they alter toxicity, mobility, solubility, and chemical transformation of metals [112, 113]. The physicochemical nature of the soil, pH, moisture, microbe population, metabolic activity, type, and concentration of metal are parameters that affect interaction.  Table 7 categorizes plant–microbe symbiotic phytoremediation mechanisms involved in the uptake and removal of heavy metal contaminations.

There are several mechanisms that govern bioaccumulation, volatilization, and uptake of toxic heavy metals through plant–microbes' symbiosis. Heavy metal tolerant–plant growth promoter (HMT-PGP) microbes process uptake and removal of heavy metals via acidification, complexation (chelation), sedimentation (precipitation), and redox reactions. Reports envisaged that the acid condition of soil facilitates the adsorption and bioavailability of heavy metals in the region of rhizospheres [114]. The presence of diverse colonies of bacteria and fungi produces organic acids and chelating agents as metabolites (gluconic acid, oxalic acid, malic acid, and acetic acid) that assist solubilization of heavy metals in the soil system [115]. *Beauveria caledonica*, a heavy metal–tolerant bacterium, oversecretes oxalic acid and citric acid that solubilizes Cd, zinc, Pb, and Cu metals in the soil. Similarly, filamentous hyphae consisting of mucorrhizal fungi deeply penetrate the soil, adsorb heavy metals, and produce oxalate crystals that detoxify the intensity of heavy metals via immobilization found there [112]. Further, oxalate crystals are released by wood-rotting fungal species such as *Ganoderma aff. steyaertanum* and *Fomitopsis cf. meliae* transform highly toxic sulphate and nitrate salts of zinc, Cd, Cu, and Pb into less toxic oxalate hydrate forms [116]. The release of plant root exudate affects soil bioavailability, mobility, and bioremediation of heavy metals. It comprises enzymes, protons, organic compounds, amino acids, phytochelatins, and nutrients required for microbial colonies that reciprocate plant growth, health, and survival [117]. The release of plant enzymes and protons from root exudate assists in the perusing of acidification and electron movement in the plant–microbe zone that amplifies metal bioavailability there. For instance, Pb is phytoremediated through plant exudate-assisted *Sedum alfredii* microorganisms [118]. Further, microbially oriented redox reactions are also involved in the process of transformation of heavy metals into simpler, nontoxic forms. The presence of outer membrane c-type and porin-cytochrome protein complexes in the bacteria (species of *Geobacter* and *Shewanella*) executes the microbial heavy metal reduction process [119]. Moreover, a few specific enzymes such as multicopper oxidase oxidize Cu, chromate reductase reduces Cr, and protein MerA mediates the reduction of Hg metal into less toxic forms, thus aiding in the phytoremediation process. *Cellulosimicrobium cellulans* and species of *Bacillus*/*Geobacillus* enhance bioaccumulation and oxidation of heavy metals (Cr and As) contaminated soil via bioaccumulation at the roots and shoots of plants [120]. Metal-binding peptides, that is, glutathione-derived peptides and mettalothioneins (MTs) of the rhizospheric microbes, are produced in the heavy metal–stressed environment and are involved with the binding of heavy metals via the process of chelation. MTs are cysteine-enriched peptides and have a high affinity for Hg, Cu, and Cd metals. *Bacillus cereus* and *Providencia vermicola* (SJ2A) are reported for increased biosynthesis of MT in their cytoplasm when exposed to a Pb-concentrated environment [121, 122]. Similarly, *Glomus intraradices* or *Rhizophagus irregularis* exhibits vacuolar compartmentalization of zinc, Cd, and Cu in the extraradical mycelium and contributes to symbiosis with clover [123]. Rhizobia, diazotropic bacteria, are well-known legume growth enhancers and can survive in heavy metal–contaminated soil. It exhibits noble examples of plant-microbe symbiosis. Diverse varieties of plant growth promoter traits of rhizobia and mycorrhiza are engaged in performing various functions such as nitrogen fixation, solubilization of insoluble heavy metals, and production of siderophores and phytohormones. Chemicals including 2,3-butanediol and acetoin facilitate rhizobia for detoxification of heavy metal–polluted soil.

Phytoremediation is a very slow process and takes several months or years for heavy metal remediation. Further, all plants are not supportive of the removal of specific heavy metals.

## 6. Conclusion

Heavy metal contamination has become a potential threat to the environment and food chain. The current scenario requires an economical and ecofriendly heavy metal bioremediation approach for the active removal via microorganisms. The study presents significant contributions of genetically modified microorganisms that are potentially efficient for the biosorption and bioaccumulation of toxic metal pollutants from the environment. They present attractive opportunities in the industrial waste management sector owing to their exclusive mechanism of actions which involve different importers or channels for effective uptake and mineralization of toxic heavy metals, although the selection of appropriate strains of microbes, suitable biosorbents, selective heavy metal targeting, and effective operating conditions are still challenges for the bioremediation of heavy metal pollutants. Novel pathways of metabolism are required to develop genetically modified microorganisms that efficiently bioremediate toxic pollutants and enhance their catalytic potential. Further, DNA replication competence, the capability of used vectors to carry imported DNA, and energy requirement for the preparation of molecules are other issues with these modified microorganisms. Their survival in variable ecological sites, altered biochemical pathways, and postrelease monitoring of genetically engineered microbes in the environment are also confronting. Additionally, ecological parameters and regulatory issues restrict the frequent implementation of genetically modified microorganisms in particular regions. Hence, a stringent framework and protocols are required for the assessment and monitoring of the functions of genetically modified microorganisms.

## Figures and Tables

**Table 1:** Various anthropological sources and permissible limits of heavy metals, a significant polluter of the environment [9, 10].

**Category of class**	**Heavy metal**	**Permissible limit in India (mg/L)**	**Permissible limit in USPEA (mg/L)**	**Anthropogenic sources**
Macronutrients	Iron	0.3	0.3	Metal refining
	Cobalt			Mining (cobalt arsenide)
	Aluminium	—	0.2	Industrial aluminium production

Micronutrients	Copper	0.05	1.3	Plastic and electroplating
	Nickel	0.05	0.1	Galvanization, paint, and battery
	Chromium	0.05	0.05	Leather industry and electroplating industry
	Manganese	0.1	0.002	Mining, burning fossil fuels, and pesticides

Toxic heavy metals	Cadmium	0.01	0.005	Paint, galvanized pipe, and pesticides
	Mercury	0.001	0.002	Combustion of coal, cement, and nonferrous metal production
	Arsenic	0.05	0.05	Automobile industries
	Lead	0.1	0.1	Fuel combustion, pesticides, gasoline, and batteries

Radionuclides	Uranium	—	30 *μ*g/L	Monazite sands and uranium ore mine
	Thorium	—	15 pCi/L	Thorium mine and ceramic industries
	Radium	—	5 pCi/L	Milling of uranium and deep bedrock aquifers

**Table 2:** Myriad sophisticated analytical tools for the detection of heavy metals in plants.

**Type of analysis**	**Detection method**	**Principle**	**Merits**	**Limitations**	**Ref.**
Quantitative analysis	Atomic absorption spectrometer	It provides both qualitative and quantitative analyses via absorption of distinctive spectral lines generated from specimen	It is used to detect various heavy metals such as silver, chromium, copper, cadmium, calcium, and mercury accumulated in diverse parts of plants. It is the most popular and potent analytical tool for elemental analysis owing to be economic and better repeatability	It is unable to provide details of chirality and chemical structure and cannot do nonelemental analysis	Preetha and Kartha [33]
	Inductively coupled plasma–mass spectrometry	It detects trace/heavy metals by assessing light emission with specific *λ*max on exposing the electric current	It has the potential to detect versatile and multiple heavy metals at very low concentrations in a limited time. It is very sensitive and can identify isotopes	Expansive and required high pure gases; interference should be controlled	Penanes et al. [34]

Chemical form analysis	Atomic fluorescence spectrometry	It is based on emission spectroscopy. The emitted fluorescence is transformed into an electrical signal that is detected by a photoelectricity detector	It offers high accuracy, better sensitivity, and concurrent analysis of multiple heavy metals. Its operating cost is low and requires less sample doe detection. It detects ultratrace element and their chemical forms in plant	Slow process and can only identify one element at a time; few samples show interference with fluorescence	Peng et al. [35]
	X-ray absorption spectroscopy	It utilizes synchrotron X-ray sources that differ in photon energy to analyse the absorption coefficient of a specimen. X-rays excite core electrons in the form of waves which disseminate surrounding atoms	It requires a smaller acquisition time and exhibits high sensitivity to analyse samples without damaging their chemical nature. It identifies heavy metals, coordination numbers, and their chemical forms faster than other tools	Penetration of sample is limited and does not provide sensitive detection limits of heavy and trace metals	Ren et al. [36]

Spatial distribution	Laser ablation-inductively coupled plasma–mass spectrometry	It works via melting and vaporizing of the specimen through laser ablation that causes a photothermal effect on the sample surface and forms a tiny burrow hole. Further analyzed by mass spectrometry that detects the type and distribution of heavy metals	It offers simultaneous estimation of multiple metals. It is very rapid and sensitive and provides spatial distribution for both elements and isotopes at a microregional level	Exhibit isobaric interference and lack of precision	Pan et al. [37]
	X-ray fluorescence	The principal is based on the specific X-ray emission spectrum generated by the specimen	It measures both quantitative and qualitative metal distribution in plants/alga single cell, hence suitable for surface analysis of plants as it can predict the mechanism of metal uptake, that is, accumulation or absorption	Cannot be used precisely when heavy metal concentrations are below parts per million level, issue of autofluorescence	Feng, Zhang, and Peiqiang [38]

Electrochemical method	Stripping voltammetry	Measurement is based on potential, current, or charge that directly correlates concentration of heavy metals	The method uses electrodes and is applied for consecutive and qualitative/quantitative analysis of heavy and transition metals	Initially, tooling is costly, and by-products are harmful to the environment, and high power consumption	Okpara et al. [39]

**Table 3:** Impact of environmental heavy metal pollutants in the flora and fauna.

**Heavy metal**	**Source of contamination**	**Effects on microorganisms**	**Effect on plants**	**Effect on human**	**Ref.**
Arsenic	Ceramics, smelting, glass, refining of metallic ores, wood works, pesticide manufacturing, preservation, and semiconductor manufacturing	Deactivates enzymes	Damages cell membranes and inhibits root extension and growth, and loss of fertility interferes in critical. Metabolic processes and fruit production.	Causes developmental anomalies, cardiovascular and peripheral vascular disease, diabetes, hearing loss, neurologic and neurobehavioural disorders, portal fibrosis, and hematologic disorders	Gupta et al. [42] and Tchounwou et al. [43]

Lead	Through inhalation of lead-polluted dust particles and aerosols. Ingestion of lead contained food and water	Causes denaturation of proteins and nucleic acid. It inhibits transcription and enzyme activities	Anorexia, damage to neurons. It causes chronic nephropathy and high blood pressure. Cases of insomnia, reduced fertility, and damage to the renal system are revealed	Affects the nervous system and gastrointestinal tract. It causes headaches and loss of memory, and acute exposure may cause brain and kidney damage	S. Flora, G. Flora, and Saxena [44]

Thallium	Combustion of fossil fuels, cement production, and metal smelting	Affects DNA and inhibits enzyme activities and growth	Inhibits enzyme activities and reduces growth	Alopecia, ataxia, burning feet syndrome, coma, convulsions, delirium, fatigue, gastroenteritis, hair fall, hallucinations, hypotension, insomnia, nausea, and tachycardia	Babula et al. [45]

Mercury	Industrial and agricultural operations. Fish consumption	Denaturation of protein and disruption of the cell membrane. Decrease population size and check enzyme function	Influence photosynthesis, affects the antioxidative system, enhances lipid peroxidation and genotoxic effect, and restricts plant growth and yield	Decreases fertility rate; creates ataxia, deafness, and dementia; causes gastrointestinal irritation and gingivitis; and manifests kidney problems and pulmonary edema	Wang et al. [46]

Copper	Copper polishing, paint, printing operations, and mining	Disrupt cellular function and obstruct enzyme activities	Causes chlorosis and retard growth and shows oxidative stress via inhibiting enzyme activities	Causes kidney damage, abdominal pain, anemia, headache, liver disease, and metabolic disorders	Chibuike and Obiora [47]

Chromium	Dyeing, paint electroplating, tanning, and steel fabrication and textile industries	Growth inhibition and elongation of lag phase. Affects oxygen uptake	Chlorosis, wilting, and biochemical lesions. Oxidative stress and reduced biosynthesis germination	Bronchopneumonia, emphysema, chronic bronchitis, diarrhoea, headache, irritation of the skin, lung cancer, itching of the respiratory tract, renal failure, and reproductive toxicity	Cervantes et al. [48]

Cadmium	Pesticide, fertilizer, plastic, refining, mining, and welding	Denature protein, damage nucleic acid, and inhibit transcription and cell division	Chlorosis, growth inhibition, decrease in plant nutrient content, and reduces seed germination	Bone disease, headache, emphysema, coughing, hypertension, kidney diseases, lymphocytosis, lung, prostate cancer, and microcytic hypochromic anemia	Sankarammal, Thatheyus, and Ramya [49]

Antimony	Coal combustion, smelting, mining, volcanic eruption, and soil erosion	Prevents enzyme activities and reduces growth rate	Inhibits chlorophyll synthesis, checks synthesis of metabolites, and causes growth inhibition	Cardiovascular diseases, conjunctivitis cancer, nasal ulceration, respiratory and liver diseases, and dermatitis	Blais et al. [50]

Selenium	Mining and coal combustion	Inhibits growth rate	Changes in protein properties and reduces plant biomass	Gastrointestinal disturbances and dysfunction of the endocrine system. Impairs the activity of natural killer cells and damages the liver	Germ et al. [51]

Nickle	Paints, electroplating, nonferrous metal porcelain, and enamelling	Damages cell membranes, inhibits metabolic enzyme activities, and causes oxidative stress	Inhibits enzyme activities, decreases chlorophyll content, and hinders growth and nutrient uptake	Chest pain, cardiovascular diseases, dry cough dermatitis, kidney diseases, lung and nasal cancers, and shortening of breath	Fashola, Ngole-Jeme, and Babalola [52]

Zinc	Oil refinery, brass manufacturing, plumbing, and mining	Decreases in biomass and inhibits growth	Inhibits photosynthesis and growth rate and affects chlorophyll content and germination rate	Gastrointestinal irritation, ataxia, liver and kidney failure, lethargy, seizures, metal fume fever, prostate cancer, hematuria, depression, and impotence	Gumpu, Sethuraman, and Krishnan [53]

**Table 4:** Genetically modified bacteria and their expressed genes for uptake of heavy metals [25].

**Genetically modified bacteria**	**Expressed genes**	**Heavy metal uptake**
*Methylococcus capsulatus*	CrR	Chromium (Cr 6+)

*Pseudomonas* K-62	Organomercurials lyase	Mercury

Strains of *E. coli*	Metalloregulatory protein ArsR	Arsenic
	SpPCS	Cadmium
	Organomercurials lyase	Mercury

*P. fluorescens* 4F39	Phytochelatin synthase	Nickel

*Achromobacter* sp. AO22	Mer	Mercury

*Ralstonia eutropha* CH34 and *Deinococcus radiodurans*	merA	Cadmium and mercury

Strain of *P. putida*	Chromate reductase	Chromium

**Table 5:** Major transporters of microorganisms for targeting heavy metals.

**Transporter class**	**Name of transporter**	**Superfamily**	**Microorganism**	**Targeted heavy metals**
Channels	Fps1, MerT/P, and GlpF	Mer 1.A.72 and major intrinsic protein 1.A.8	*Saccharomyces cerevisiae*	Arsenic
			*Serratia marcescens* and *Pseudomonas K-62*	Mercury
			*Escherichia coli* and *Corynebacterium diphtheriae*	Arsenic

Secondary carriers	Pho84	Major facilitator 2.A.1 Transporter-opsin-G	*Saccharomyces cerevisiae*	Arsenic Arsenic
	Hxt7 and NixA/homologs	Protein-coupled receptors	*Saccharomyces cerevisiae*, *Helicobacter pylori*, *Novosphingobium aromaticivorans*	Nickel/cobalt

Primary active transporters	MntA, TcHMA3, and CopA	P-type ATPase 3.A.3	*Lactobacillus plantarum*	Cadmium
			*Thlaspi caerulescens*	Cadmium
			*Enterobacter hirae*	Copper

**Table 6:** List of microbes and their bioaccumulation capacity for heavy metals [105].

**Microorganisms**	**Heavy metals**	**Import/storage**	**Bioaccumulation capacity**
*E. coli* K-12 MG1655 *E. coli* JH109	Nickel and cobalt	Import Both	4.8 mgCo g_DW_^−1^ 6 mgNi g_DW_^−1^ 9.89 mg Ni g_DW_^−1^

*S. cerevisiae* *C. glutamicum* 13032	Arsenic	Storage	0.22 mgAs^+3^ g_DW_^−1^
		Both	2.16 mgAs^+4^ g_DW_^−1^

*E. coli* XL1 blue *Mesorhizobium* sp.	Cadmium	Import	0.068 mg Cd g_DW_^−1^
		Storage	4.047 mg Cd g_DW_^−1^

*S. cerevisiae* BY4743 *E. coli* BL21	Copper	Storage	103.3 mg Cu g_DW_^−1^
		Storage	145 mgCu g_DW_^−1^

*E. coli* *Rhodopseudomonas palustris* GIM1.167 *E. coli* JM109	Mercurial sps.	Storage	4.012 mgHg g_DW_^−1^
		Both	77.58 mg mgHg g_DW_^−1^
		Both	17.65 mgHg g_DW_^−1^

**Table 7:** Phytoremediation mechanisms for bioaccumulation and detoxification of heavy metals.

**Plant–microbe symbiotic phytoremediation mechanisms**			
**Bioactivation of metal**	**Enhanced uptake by transporters**	**Detoxification by distribution**	**Vacuole sequestration of metals**
Root microbe interaction enables the bioactivation of metals in the rhizosphere. Plants may alter their membrane permeability, may change the metal binding capacity of cell walls, and may ooze more chelating substances.	Metal transporters enhance the uptake of heavy metals across membranes through the associated protein superfamily (macrophage protein family and cation diffusion facilitator).	Detoxification is executed by way of a complex process. It involves the binding of cell walls and metal chelates in the cytoplasm via various ligands. Utilized approaches are phytochelatins, metal-binding proteins, and metallothioneins.	Here, heavy metals concentrate in the aerial parts of plants to levels far exceeding than soil. Hyperaccumulation occurs, and plants absorb high levels of contaminants either in their shoots, roots, or leaves.

## Data Availability

All data generated or analyzed during this study are available upon request from the corresponding author.
